# Efficacy of Therapeutic Exercise in Reducing Pain in Instrumental Musicians: Systematic Review and Meta-Analysis

**DOI:** 10.3390/healthcare12131340

**Published:** 2024-07-05

**Authors:** Cristina Iglesias-Carrasco, María de-la-Casa-Almeida, Carmen Suárez-Serrano, Maria-Luisa Benítez-Lugo, Esther M. Medrano-Sánchez

**Affiliations:** 1 Department of Physical Therapy, Faculty of Nursing, Physical Therapy and Podiatry, Universidad de Sevilla, 6, Avenzoar St., 41009 Sevilla, Spain; criiglcar@alum.us.es; 2 Research Group CTS305, Department of Physical Therapy, Faculty of Nursing, Physical Therapy and Podiatry, Universidad de Sevilla, 6, Avenzoar St., 41009 Sevilla, Spain; csuarez@us.es (C.S.-S.); marisabeni@us.es (M.-L.B.-L.)

**Keywords:** exercise therapy, physical activity, physical therapy, pain, instrumentalist musicians

## Abstract

Playing-related pain poses a significant health concern for musicians, often impacting their ability to perform. Therapeutic exercise emerges as a viable approach to alleviate these symptoms, offering a low-cost intervention with minimal side effects. This review seeks to examine and assess the efficacy of therapeutic exercise in reducing pain intensity among instrumental musicians. Three major databases (PubMed, Web of Science, and Scopus) were systematically searched from November 2023 to June 2024. The inclusion criteria required studies to be randomized clinical trials focusing on pain intensity in instrumental musicians, published in the last 10 years. Two independent researchers assessed the characteristics and methodological quality of the selected studies. Out of 305 identified studies, 15 underwent full-text reviews, with 5 ultimately included in the analysis. The total participant count was 273, with an average intervention duration of 32.5 min per session, twice weekly for eight weeks. Overall, therapeutic exercise interventions demonstrated favorable effects, with three studies exhibiting good methodological quality. The meta-analysis revealed significant positive results favoring exercise in reducing pain intensity, with positive responses observed across all clinical populations, so therapeutic exercise appears to be an effective approach for reducing pain intensity in musicians experiencing playing-related pain.

## 1. Introduction

Pain-related musculoskeletal disorders (PRMD) associated with musical practice encompass a spectrum of symptoms that hinder a musician’s ability to perform at their accustomed level, including pain, weakness, numbness, tingling, and other impediments [1,2]. These symptoms range from mild discomfort and muscle fatigue to more severe conditions, such as focal dystonia [3,4,5,6] and chronic pain, ultimately impeding practice and performance [1].

Presently, chronic pain affects 80% of musicians over the course of their careers, with an incidence rate 60% higher than that of the general population, a figure exacerbated among music students [7,8,9]. As a risk factor, a greater number of injuries are associated with string or percussion instrument musicians, with wind instruments causing the fewest injuries [10]. The shoulder, neck, and upper spine were common sites of injury, with most injuries being chronic, recurrent, and preventable.

The etiology of this pain primarily stems from the rigorous physical and psychosocial demands imposed on musicians [11]. These demands precipitate functional and structural alterations in the brain, including enlargement of the corpus callosum [12,13,14], deepening of the central sulcus, and thickening of the primary motor cortex [15], exacerbated by risk factors such as anxiety, repetitive movements, and extensive practice [1,2,15,16,17]. Consequently, early education on pain management and optimal physical conditioning are vital for musicians’ well-being and longevity in their craft [1,2,9,18,19].

Playing a musical instrument entails a complex multimodal task that engages higher-order brain structures, necessitating integration across auditory, somatosensory, motor, and cognitive domains, facilitating neuroplastic changes and skill acquisition [20,21,22,23,24]. Dysfunction in this intricate system can lead to somatosensory cortical reorganization, heightening pain sensitivity, lowering pain thresholds, and exacerbating pain, particularly in individuals with elevated anxiety levels and stage fright [16].

Despite acknowledgment from various authors regarding the importance of maintaining good physical condition and incorporating therapeutic exercise (TE) into musical practice [19,22,23,24] and advocating for greater emphasis on TE in PRMD prevention and treatment [24,25], there remains a dearth of specific exercise programs tailored to improve musicians’ physical well-being. Moreover, scientific evidence supporting the efficacy of TE in this context is limited.

Therefore, the objective of this systematic review with a meta-analysis is to evaluate the effectiveness of TE in alleviating pain intensity among instrumental musicians.

## 2. Materials and Methods

A systematic review of randomized controlled clinical trials was carried out, accompanied by a meta-analysis following the standards described in the PRISMA statement [26]. The systematic review was registered in the International Prospective Registry of Systematic Reviews (PROSPERO), and its registry identifier number is CRD42020191590.

The main variable of the study was pain intensity, measured by any of the scales validated for that purpose. Pain interference was defined as a secondary variable and refers to the degree to which pain causes limitation, hindering, or interferences with physical, cognitive, emotional, social, or recreational activities, as well as sleeping and enjoyment of life [27]. This variable was measured by the Pain Intensity and Interference Questionnaire for Professional Orchestra Musicians [28], which includes items from the Brief Pain Inventory (BPI) [29] to measure pain intensity, and items from other questionnaires such as Arm, Shoulder and Hand Disabilities (DASH) [30], and Musculoskeletal Pain for Musicians (MPQM) [31] to measure pain interference.

### 2.1. Data Sources and Searches

Bibliographic searches were carried out between November 2023 and June 2024, and the Web of Science (WoS), PubMed, and Scopus databases were used. Descriptors included in the medical subject headings (MeSH) were: “exercise therapy”, “physical activity”, “physical therapy” and “pain”. Furthermore, these descriptors were also combined with the terms “musicians”, “instrumentalist musicians”, “performing artists”, and “controlled trial”. Different combinations were made using the Boolean operators AND and OR. The search strategies are provided as Supplementary Material.

### 2.2. Study Selection

Included studies had to be clinical trials whose main variable was pain intensity in instrumental musicians, published in the last 10 years, which included physical activity and/or ET as therapy at least in one of the intervention groups, and the studies were published in English or Spanish.

### 2.3. Data Extraction and Quality Assessment

Two researchers independently examined the characteristics (participants, intervention, and outcomes) of the selected studies and assessed the methodological quality. Discrepancies were resolved by consensus, and only when this consensus was not achieved, the participation of a third assessor was requested. For the assessment of the methodological quality and the risk of bias of the studies included in this review, the PEDro [32] scale was used based on the Delphi list, with scores from 0 to 10, of which scores of 9–10 are related to excellent methodological quality, between 6–8 have good methodological quality, between 4–5 moderate methodological quality, and, below 4 points, poor methodological quality [33,34].

### 2.4. Data Synthesis and Analysis

Regarding the synthesis of the results and the meta-analysis, those qualitative aspects of the studies were described in a narrative way, and the results were grouped when possible. The quantitative synthesis (meta-analysis) was carried out using the Review Manager software (RevMan) [Computer program], version 5.4 (The Cochrane Collaboration, 2020) to summarize the effects, and forest diagrams were obtained to show the results.

The standardized difference of means was calculated with a confidence level of 95% because each study used different instruments to measure the same variable.

Regarding the evaluation of heterogeneity, a visual inspection of the forest plot and the value of I^2^ was carried out. According to the interpretation guidance provided by Deeks and Higgins [35], while I^2^ test results ranging from 0–40% may not report relevant levels of heterogeneity, those of 30–60% may indicate moderate heterogeneity and between 50–90% substantial heterogeneity.

## 3. Results

**Figure 1:**
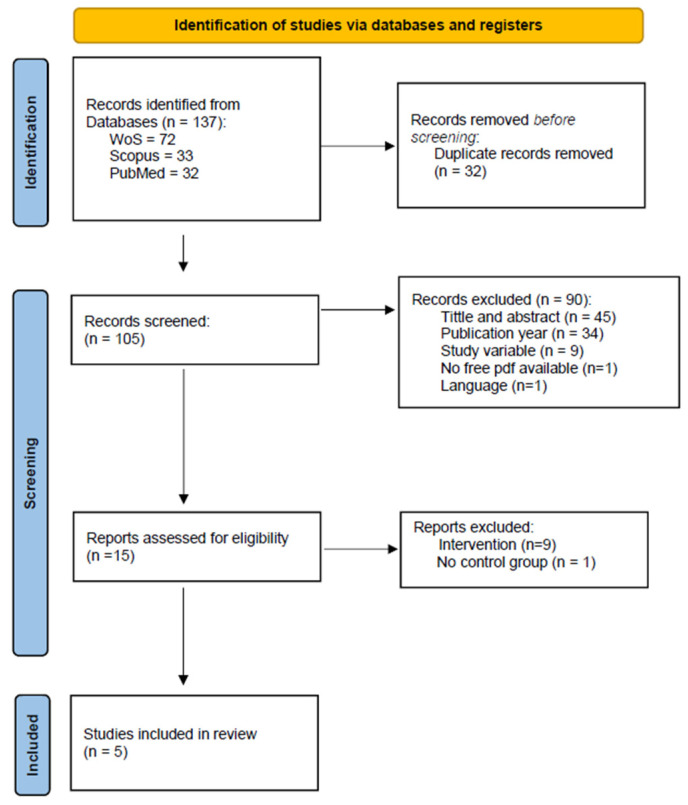
PRISMA flow diagram.

In the study selection process, a total of 305 articles were initially identified, of which 15 studies were selected for the full-text review, and after reading them, 5 studies conformed to the results of the review (Figure 1. PRISMA Flow Diagram). The main characteristics of the included studies are shown in Table S1 (available as Supplementary Materials).

### 3.1. Qualitative Synthesis of the Results

The assessment of methodological quality using the PEDro [32] scale showed three studies of good methodological quality, obtaining scores of 8 [36], 7 [37], 6 [38], and 4 [38,39], respectively (Table S2, available as Supplementary Materials).

Health education programs [36] based on a postural hygiene informative presentation, dynamic exercises during instrument practice, gradual adaptation of the body to workload, and physical stress or healthy lifestyle habits were some of the interventions described in the studies included in this review. Regarding the type of exercises, the authors chose specific exercises [36] for the neck, shoulder, abdominals, back and hips, as well as neck and shoulders-specific high-intensity strength training [38] (exercises with dumbbells, one-arm rowing, abduction, and shoulder elevation, and reverse fly in a 45° plane prone), as well as exercise on a bicycle/ergometer [38] at 50–70% VO2 maximum, and included individualized feedback from the teacher, self-analysis of body posture using a mirror, visualizing body posture with mirrors, and analyzing each other’s body postures [37]. This also included chair massage, strength, and mobility exercises [39] (supine and prone positions and sitting and standing), postural training, ROM (10 min), stretching (5–7 min), as well as isometric (5–10 min) and stabilization exercises (5 min) with exercise balls [40].

The frequency of training varied from two [36,39] to three [38,40] times a week, and the duration of the exercise programs ranged from 4 [39] to 9 [38] or 11 [36,37,40] weeks. The duration of sessions also varied between 20 [38] 35–45 [36,39,40] min.

Of the included studies, two measured pain intensity [38,40] using the numerical verbal and visual analog scales, two [36,39] used the pain dimension included in the Pain Intensity and Interference Questionnaire for Professional Orchestra Musicians [28], and one [37] used the DASH questionnaire [30]. In all of them, a significant decrease in pain in the experimental groups can be observed.

Roos et al. [36] analyzed the efficacy of a TE program in the experimental group compared to the absence of intervention in the control group, showing statistically significant differences between the groups in favor of the experimental one (*p* = 0.025). Baadjou et al. [37] observed that a biopsychosocial prevention course was not superior to physical activity promotion in reducing disability. Andersen et al. [38] observed that both groups were statistically significant for intensity of pain reduction (*p* = 0.05), but they did not find differences between both treatments. Anna Cyganska et al. [39] analyzed the effectiveness between chair massage and TE compared to the absence of intervention in the control group, showing significant improvements in the pressure sensitivity of the tested trigger points in the groups subjected to treatment, being the largest differences in the massage group. Finally, Serkan Usgu et al. [40] observed that both groups were statistically significant for intensity of pain reduction (*p* < 0.05).

Concerning the secondary variable of this review, pain interference, only one of the studies included assessed this aspect using the Pain Intensity and Interference Questionnaire for Professional Orchestra Musicians (MPIIQM) [24], and their results showed a significant improvement (*p* = 0.006) in the impact of pain on the quality of life of patients in the experimental group [36,39].

### 3.2. Quantitative Synthesis of the Results

**Figure 2:**
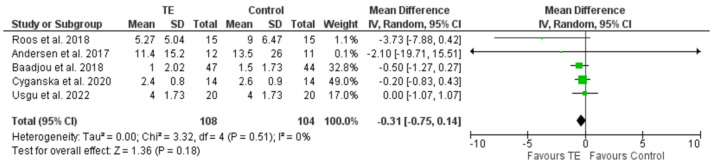
Forest plot illustrating the random effects model for the difference in pain intensity between groups after the intervention. TE: therapeutic exercise [36,37,38,39,40].

The analysis was carried out assuming a random effects model (Figure 2) in which the results of the pain intensity variable were entered in the experimental group (after the application of TE) and in the control group.

A value of zero was observed for both Tau^2^ and I^2^ (Heterogeneity: Tau^2^ = 0.00; Chi^2^ = 3.32, df = 4 (*p* = 0.51); I^2^ = 0%), considering that the clinical trials were statistically homogeneous, as well as the effects between clinical trials.

**Figure 3:**
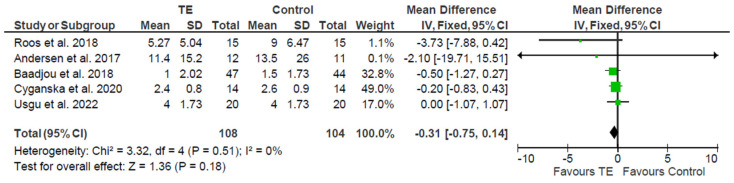
Forest plot illustrating the fixed effects model for the difference in pain intensity between groups after the intervention. TE: therapeutic exercise [36,37,38,39,40].

Therefore, the analysis was repeated, this time following a fixed effects model (Figure 3), which yielded a significant difference between the control and experimental groups, with the favorable group being the experimental or intervention group.

A reduction in pain intensity was observed with the applied treatment, both in the comparison of post-intervention pain levels and in the comparison of the difference in these levels before treatment and after treatment (mean difference equal to −0.31 in an interval from −1.07 to 1.07, with a confidence level of 95%) showing that pain levels after the intervention were lower in the experimental group than in the control group, with differences in pain intensity pre- and post-intervention.

## 4. Discussion

The results of this review show that TE significantly reduced the pain intensity compared to the control groups where other interventions or no interventions were carried out.

However, the fact that there are very few studies investigating the benefits of TE in instrumental musicians makes us cautious about the observed results, which reflect the need for further research in this field.

On the other hand, regarding the methodological quality measured with the PEDro scale [32], we found studies of good methodological quality according to Sherrington et al. [32,33], items 5 (blinding of all subjects), 6 (blinding all therapists who administered therapy), and 7 (blinding all assessors who measured at least one key outcome, respectively) being the ones with a lower scoring due to the poor masking of the participants, therapists and/or evaluators, respectively. This concern is frequently observed in studies that compare exercise programs for their complexity to mask the participants or the physical therapists or evaluators due to the nature of the intervention. Therefore, taking into consideration this difficulty, we interpret that the methodological quality of all the studies was good or even excellent in the case of Roos et al. [36].

In the same context, according to the GRADE System [41] (classification of the quality of evidence and strength of the recommendation of the studies), the level of evidence of the studies included in our review corresponds to a moderate level of scientific quality and a strong grade of recommendation [41,42]. Despite the fact that the randomized clinical trials included were homogeneous and the positive results were in favor of TE, demonstrated in the meta-analysis, the number of studies included was small, and the number of subjects was moderate (ranging between 23 and 273 participants), which may decrease the external validity of the studies.

Regarding the variable “pain intensity” after the application of TE, the qualitative synthesis of the results of the five studies suggests greater effectiveness in the experimental group than in the control group, showing a significant decrease in pain in the groups of patients who performed TE programs, which seemed to have major positive effects on the pain levels between groups [36,37,38,39,40].

Anna Cyganska et al. [39] found that chair massage, in terms of pressure sensitivity of trigger points, showed the largest differences between both groups subjected to treatment. This could be due to the relaxation and calmness of the sympathetic nervous system. This is a relevant issue to consider in future research because it is a good form of prophylaxis that can be performed at the workplace or residence of musicians.

On the other hand, one of the studies [40] showed that a similarly structured therapeutic exercise program had positive effects on pain, functionality, posture, and social roles in string and woodwind musicians. However, it suggested considering instrument-specific and physical requirements when prescribing exercises to musicians to address the particular demands of each instrument.

Unlike pain intensity, pain interference was only evaluated by Roos et al. [36], observing a statistically significant difference in the experimental group. Despite the fact that TE was shown to be effective in improving this variable, the fact that it is the only existing study that has analyzed this variable, and due to the drawback of its small sample size, makes us cautious when generalizing the results. However, if TE-specific programs are effective in reducing pain in instrumental musicians, this would have an important impact on their quality of life, which is undoubtedly an interesting point for future research.

Other systematic reviews [43,44,45] and clinical practice guidelines [46] suggest the existence of strong evidence of the efficacy of TE in the treatment of musculoskeletal pain, as suggested by our results. Specifically, there is a recent systematic review about prevention [47], which includes studies with TE interventions for pain in musicians like ours but is not exclusive to randomized control trials. In addition, its main objective is not to determine the effectiveness of TE in the intensity of pain in instrumental musicians but rather to contemplate any intervention that improves pain in this population.

Improvement in pain in musicians after the implementation of TE programs was already observed by Foxman et al. [22] in 2006, who highlighted the importance of having good physical condition in these populations and how TE had not been taken into account in music schools and conservatories despite being one of the essential mainstays for the prevention and improvement of musculoskeletal dysfunctions due to instrumental practice.

In more recent years, other authors have also claimed the importance of TE in music schools and conservatories as the first line of prevention of pain related to musical practice [23,48]; however, as our review shows, the number of studies investigating its efficacy is still very small, and therefore, scientific evidence in this regard is low.

We found other studies [19,49,50] that, despite not being randomized clinical trials, observed improvements in pain in instrumental musicians after the application of TE, but, similar to the studies included in this review, all of them presented small samples.

Therefore, we would like to remark on the need to develop more research in this field as a perspective of our review, which should include the differentiation of a specific guideline depending on the instrument since each instrumentalist requires physical rehabilitation and specific training of the muscle groups involved in the execution of each type of instrument. An assessment of the degree of effect on the quality of life and pain interference in this population should be included, given the effect that these musculoskeletal disorders have on many musicians.

### Limitations

Regarding the limitations of this review, it should be noted the scarce number of clinical trials that have tested the efficacy of therapeutic exercise in reducing pain in instrumental musicians, so very few studies have been included in this meta-analysis.

## 5. Conclusions

Based on the results of the review and meta-analysis, we can suggest that TE seems to be an effective therapeutic option to reduce the intensity of pain in instrumental musicians, as well as its effectiveness on the quality of life of the subjects.

However, further research is needed to bring stronger evidence of its effectiveness.

## Data Availability

Not applicable.

## Supplementary Materials

The following supporting information can be downloaded at: https://www.mdpi.com/article/10.3390/healthcare12131340/s1, Table S1. Characteristics of the studies included in the review. Table S2. Assessment of methodological quality using the PEDro scale.
