# Supplementary material for: Efficacy of Therapeutic Exercise in Reducing Pain in Instrumental Musicians: Systematic Review and Meta-Analysis
Source: Healthcare (Basel). 2024 Jul 5;12(13):1340. doi: 10.3390/healthcare12131340 (PMC11241052; doi:10.3390/healthcare12131340)
Supplement: Supplementary file 1 [file healthcare-12-01340-s001.zip › healthcare-3023564-supplementary.pdf]

SEARCH STRATEGY

WoS

(“physical therapy” OR “exercise therapy”) AND (musicians OR instrumentalist) AND pain → ***72 results***

Scopus

(“exercise therapy” OR “physical therapy”) AND (musicians OR instrumentalists) AND pain →***33 results***

PubMed

(“exercise therapy” OR “physical therapy”) AND (musicians OR instrumentalists) AND pain → ***32 results***

Table S1. Characteristics of the studies included in the review.

Study	PEDro Score	Participants	Intervention	Study variables	Measurements	Outcomes
**Marianne Roos et al.[36]**<br><br>**2018, Quebec (Canada)**	8/10	30 students and professional orchestra musicians with or without Playing-related Musculoskeletal Disorders (N=30)<br><br>**Experimental:** 15<br>Age: 36.8±11.5<br><br>**Control:** 15<br>Age: 39.3±12.7	**Experimental:**<br>40-minute educational presentation (muscles involved, importance of physical activity to prepare for the demands, management and adaptation to stressors, and exercise program instructions) + exercise program consisting of warm-up (5 min), series of 6 exercises each one of increasing difficulty in the neck, shoulder, abdominal, back and hip regions (25-30 min) + cool down (5 min). Duration of 35-40 min per week (including 5 min warm-up and cool-down).<br><br>**Control:**<br>Non-intervention and continuation of their basic activities of their daily living.<br><br>**Duration:** 2 times/week for 11 weeks.	Musculoskeletal Pain Intensity and Interference Questionnaire for Profesional Orchestra Musicians (MPIIQM)<br><br>Intensity (0-40) and pain impact (0-50).	Measurements at the beginning (week 1) and at the end (week 11) of the exercise program.	• Clinically and statistically significant improvements in pain intensity and pain impact.<br><br>**•Pain Intensity**<br>Experimental group<br>Pre-Intervention → 9.33±5.89<br>Post-Intervention → 5.27±5.04<br>- Change from the beginning: p=0.01 → $p<0.05$.<br><br>Control group<br>Pre-Intervention → 8.60±4.75<br>Post-Intervention → 9.00±6.47<br>- Change from the beginning: p=0.763 → $p>0.05$.<br><br>**•Pain Impact**<br>Experimental group<br>Pre-Intervention → 12.00±10.56<br>Post-Intervention → 3.00±4.66<br>- Change from the beginning: p=0.006 → $p<0.05$.<br><br>Control group

						Pre-Intervention → 7.47±6.79<br>Post-Intervention → 7.67±11.60<br>- Change from the beginning:<br>p=0.933 → p>0.05.<br><br>• Statistically significant improvements in the experimental group compared to the control group (p= 0.028 → p<0.05).
**Baadjou et al.[37]**<br><br>**2018, Maastritcht (The Netherlands)**	7/10	136 first-year bachelor students from the aca- demic year 2012–13 and first- and second-year students from the aca- demic year 2013–14<br><br>**Experimental:** 68<br>Age: 20 (19-22.25)<br><br>**Control:** 68<br>Age: 20 (19-22.25)	**Experimental:**<br>Standardized PowerPoint presentation, semi-structured class discussions, peer model stories video of role model with physical complaints, individualized feedback from teacher on body posture, analysing own body posture with mirror, individual feedback on performance posture from teacher, visualizing own body posture by use of mirrors and analysing each other's body postures.<br><br>**Control:**<br>Video on general physical activity, video on musician-specific benefits of physical activity, assignment on calories and physical activity, semi-	Pain Disability Index (PDI)<br><br>Pain: 0 (no disability) – 10 (total disability)	Measurements at the beginning (week/ T0), 10 weeks (T1), 20 weeks (T2), post-treatment (T3), 16-month follow-up (T4) and 24-month follow-up (T5).	• No significant differences between groups.<br><br>•**Pain Disability**<br><u>Experimental group</u><br>Baseline → 2 (0–10)<br>Post-intervention → 1 (0–7)<br>24-month follow-up → 1 (0–8)<br><br>•Baseline (β/OR=0.73), post-intervention (β/OR=1.41) and 24-month follow-up (β/OR= −1.0)<br><br><u>Control group</u><br>Baseline → 2 (0–7.75)<br>Post-intervention → 1.5 (0–6)

			structured class discussion, visualizing current steps with graphs, goal setting; step logbooks; visualizing step counts with graphs, and semi-structured class discussion; count steps of frequent routes.<br><br>**Duration:**<br>Experimental group had 11 classes during one year and control group had 5 classes during one year.			24-month follow-up → 4 (0–9)
**Andersen et al.[38]**<br><br>**2017, Odense (Denmark)**	6/10	23 musicians from the Odense Symphony Orchestra.<br><br>**Experimental:** 12<br>Age: 44.7±11<br><br>**Control:** 11<br>Age:47.2±7.8	**Experimental:**<br>Specific high intensity strength neck and shoulder exercises.<br>5 exercises with dumbbells, one-arm row (45º flexion with hand and knee on a flat surface), shoulder abduction (standing), shoulder elevation (standing), reverse fly (45º prone position on the bench). All exercises performed with concentric and eccentric contractions. In each session, 3 of the 5 exercises were performed in 3 series per exercise, the elevation being the only one that was repeated in each session.<br><br>**Control:**	Visual Analog Scale (VAS:0-100mm)<br><br>Pain: no pain – worst pain imaginable	Measurements at the beginning (week 1) and at the end (week 9) of the program.	• Significant improvements in the intensity of pain and in the working condition of musicians.<br><br>•**Pain Intensity**<br><u>Experimental group</u><br>Pre-Intervention → 26.3<br>Post-Intervention → 11.4<br>- Change from the beginning: p=0.05 → p<0.05.<br><br><u>Control group</u><br>Pre-Intervention → 19.7<br>Post-Intervention → 13.5<br>- Change from the beginning: p=0.09 → p>0.05.<br><br>• No significant improvements between groups (p=0.29 → p>0.05)

			Exercise on a bicycle / ergometer at 50-70% VO2 max. The subjects were in an upright position with their back at 90º with respect to the ground without holding onto the handlebars. It was emphasized that the shoulders should be relaxed so that the body weight rested in the gluteal area. The saddle was vertically aligned, and the height adjusted so that the knees were slightly bent when the pedal reached its lowest point.<br><br>**Duration:** 20 minutes 3 times / week for 9 weeks.			
**Anna Cyganska et al.[39]**<br><br>**2020, Warsaw (Poland)**	4/10	44 students of The University of Music who played a musical instrument for a minimum of 5 years.<br><br>**Massage:** 16<br>Age: 20.6±1.2<br><br>**Exercise:** 14<br>Age: 21.07±2.0<br><br>**Control:** 14	**Esperimental:**<br>**Massage:**<br>Chair massage consisting of stroking, kneading, stretching combined with swaying and passive movements of the upper limbs, and final stroking combined with slapping and shaking percussion, vibration.<br><br>**Exercise:**<br>Sitting in a chair:<br>- Anteversion and retroversion of the pelvis-20 repetitions.	Visual Analog Scale (VAS:0-100mm)<br><br>Pain: no pain – worst pain imaginable<br><br>Musculoskeletal Pain Intensity and Interference Questionnaire for Profesional Orchestra	Massage and exercise groups measurements before and after each treatment). Control group 2 measurements, 4 weeks apart.	• Significant improvements in the pressure sensitivity of the tested trigger points in the groups subjected to treatment, being the largest differences in the massage group.<br><br>No differences between men and women due to the pain intensity (12.93±5.79; p=0.51) or the pain influence (18.5 ± 11.8; p = 0.47)

Age: 20.1±1.1	- Alternating trunk twists right and left-10 repetitions.<br>- Lifting and lowering the shoulder girdle-20 repetitions.<br>- Backward shoulder girdle rotation-10 repetition.<br>- Alternating pro-and supination of the forearms-10 repetitions.<br>- Rotation in the wrist joints-10 repetitions).<br><br>Supine position:<br>- Lifting the trunk and upper limbs with simultaneous retraction of shoulder girdle-30 repetitions.<br><br>Prone position:<br>- Thigh extension in hip joint-20 repetitions per side.<br>- The bridge exercise-30 repetitions.<br>- The dead bug exercise-30 repetitions per side.<br>- Hamstring stretching-3 repetitions (30s per side).<br><br>Standing position:	Musicians (MPIIQM)<br><br>Intensity (0-40) and pain impact (0-50).

			- Stretching of the thoracic girders, resting one upper limb on the door frame-3 repetitions (30s per side).<br><br>**Control:**<br>No intervention.<br><br>**Duration:**<br>Massage group had chair massage 15min 2 times/week and exercise group had exercises 15min 2 times/week.			
**Usgu et al.[40]**<br><br>**2022, Gaziantep (Turkey)**	4/10	40 musicians aged between 18 and 65 years with persistent nonspecific neck pain in the previous 3 months.<br><br>**Violin:** 20<br>Age: 26.5 (18-63)<br><br>**Ney:** 20<br>Age:27.5 (19-51)	**Experimental:**<br><br>Postural training, ROM (10 min), stretching (5–7 min), isometric (5–10 min) and stabilization exercises (5 min) with exercise balls (diameter of 18 cm, weight of 90g and a load carrying capacity of up to 136 kg, made of soft elastic material).<br><br>**Control:**<br><br>The same exercise ball and stabilization exercises were performed.	Visual Analog Scale (VAS:0-100mm)<br><br>Pain: no pain – worst pain imaginable		• Significant improvements in the intensity of pain ($p<0.05$).<br><br>•**Pain Intensity**<br><u>Violin group</u><br>Pre-Intervention → 6 (4-9)<br>Post-Intervention → 4 (0-6)<br>- Change from the beginning: p=0.002<br><br><u>Ney group</u><br>Pre-Intervention → 6 (4-8)<br>Post-Intervention → 4 (0-6)<br>- Change from the beginning: p=0.002

	**Duration:**<br>40-45min exercise sessions three times/week during eight weeks.	• No significant improvements between groups (p=0.607 → p=0.497).

Table S2. Assessment of methodological quality using the PEDro scale

	Marianne Roos et al.[36]	Baadjou et al.[37]	Andersen L.N et al.[38]	Anna Cyganska et al.[39]	Usgu et al.[40]
**Selection criteria**	Yes	Yes	Yes	Yes	Yes
**Random allocation**	Yes	Yes	Yes	No	No
**Concealed allocation**	Yes	Yes	Yes	No	No
**Similar groups**	Yes	Yes	Yes	Yes	Yes
**Blinding of all subjects**	No	No	No	No	No
**Blinding of all therapists**	No	No	No	No	No
**Blinding of all assessors**	Yes	Yes	No	No	No
**Follow-up of more than 85% of the subjects initially allocated**	Yes	No	Yes	Yes	Yes
**"Intention to treat"**	Yes	Yes	Yes	No	No
**Statistical comparisons between groups**	Yes	Yes	Yes	Yes	Yes
**Measures of variability**	Yes	Yes	No	Yes	Yes
**Total score**	8/10	7/10	6/10	4/10	4/10
**Qualitative score**	Good	Good	Good	Moderate	Moderate
